# Odefy -- From discrete to continuous models

**DOI:** 10.1186/1471-2105-11-233

**Published:** 2010-05-07

**Authors:** Jan Krumsiek, Sebastian Pölsterl, Dominik M Wittmann, Fabian J Theis

**Affiliations:** 1 Institute for Bioinformatics and Systems Biology, Helmholtz Zentrum München, Ingolstädter Landstrasse 1, 85764 Munich-Neuherberg, Germany; 2 Department of Mathematics, Technische Universität München, Boltzmannstrasse 3, 85748 Garching, Germany

## Abstract

**Background:**

Phenomenological information about regulatory interactions is frequently available and can be readily converted to Boolean models. Fully quantitative models, on the other hand, provide detailed insights into the precise dynamics of the underlying system. In order to connect discrete and continuous modeling approaches, methods for the conversion of Boolean systems into systems of ordinary differential equations have been developed recently. As biological interaction networks have steadily grown in size and complexity, a fully automated framework for the conversion process is desirable.

**Results:**

We present *Odefy*, a MATLAB- and Octave-compatible toolbox for the automated transformation of Boolean models into systems of ordinary differential equations. Models can be created from sets of Boolean equations or graph representations of Boolean networks. Alternatively, the user can import Boolean models from the CellNetAnalyzer toolbox, GINSim and the PBN toolbox. The Boolean models are transformed to systems of ordinary differential equations by multivariate polynomial interpolation and optional application of sigmoidal Hill functions. Our toolbox contains basic simulation and visualization functionalities for both, the Boolean as well as the continuous models. For further analyses, models can be exported to SQUAD, GNA, MATLAB script files, the SB toolbox, SBML and R script files. Odefy contains a user-friendly graphical user interface for convenient access to the simulation and exporting functionalities. We illustrate the validity of our transformation approach as well as the usage and benefit of the Odefy toolbox for two biological systems: a mutual inhibitory switch known from stem cell differentiation and a regulatory network giving rise to a specific spatial expression pattern at the mid-hindbrain boundary.

**Conclusions:**

Odefy provides an easy-to-use toolbox for the automatic conversion of Boolean models to systems of ordinary differential equations. It can be efficiently connected to a variety of input and output formats for further analysis and investigations. The toolbox is open-source and can be downloaded at http://cmb.helmholtz-muenchen.de/odefy.

## Background

The ultimate goal of the increasingly popular systems biology approach is the integration of many different molecular interactions into extensive computer models that closely reflect real-life behavior of their underlying biological systems. Mathematical implementations of various biological systems have been proposed recently, including cell cycle control in yeast [1] and *Caulobacter crescentus *[2], and circadian rhythms of *Arabidopsis thaliana *[3] to name but just a few. Such studies are primarily designed to match known measurable phenotypes of the respective systems and reveal insights into the precise quantitative evolution of biochemical species over time. With a reasonable *in silico *implementation of a biological system at hand, predictions of knockout and perturbation effects can be performed by the computer.

For most biological systems, however, only qualitative information about regulatory interactions is available, which is not sufficient to implement precise kinetic rate laws for each biochemical reaction. A well-established workaround for this lack of information is the application of discretized modeling approaches. In Boolean methodology, for example, we abstract from actual molecule quantities and assign each player in the system the state *on *or *off *(e.g. active or inactive). Despite the simplicity of Boolean models we still assume them to provide information about the general dynamics and capabilities of the underlying system. Recently proposed Boolean models include developmental processes in *D. melanogaster *[4], the regulation of the mammalian cell cycle [5], the activation of T-cells [6] and EGFR signaling in human hepatocytes [7].

In [8] we described a novel technique called *HillCube *for the automatic transformation of Boolean models into systems of autonomous first-order ordinary differential equations (ODEs). HillCubes are based on multivariate polynomial interpolation and incorporate Hill kinetics (see Implementation), which are known to provide a good generalized approximation of the synergistic dynamics of gene regulation [9,10]. Important properties of the system like steady-state behavior are preserved during the transformation. Our methodology allows to enrich Boolean models built up from coarse information by features of quantitative models, such as intermediate expression levels, continuous transitions and different time-scales. Other approaches for the analysis of purely phenomenological regulatory networks have been developed recently (cf. e.g. [6,11]) but do not employ continuous, quantitative modeling.

**Figure 1:**
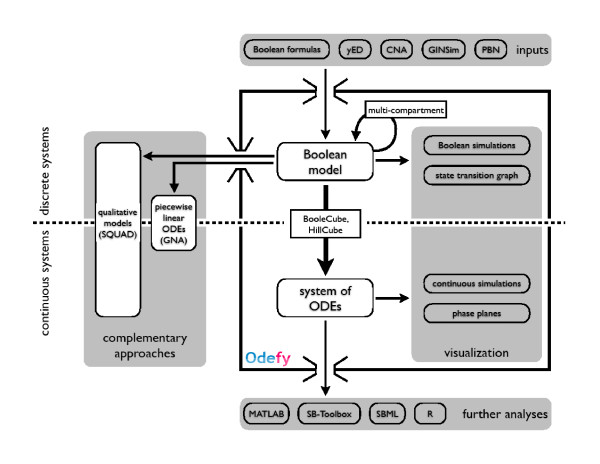
**Odefy overview**. Odefy generates models from sets of Boolean equations or Boolean hypergraphs created with yEd. Alternatively, Boolean models can be imported from the CellNetAnalyzer, GINsim or the PBN toolbox. Odefy contains a method for the automatic generation of multi-compartment models from a given single cell model. Boolean models can be exported to other discrete input formats (for the GNA and SQUAD toolboxes), used for Boolean simulations and analysis within Odefy, or they can be converted to systems of ordinary differential equation (ODE). These ODE systems can either be directly simulated and analyzed with Odefy or exported to well-established model formats, including MATLAB script files, SBML, SB Toolbox models and R script files.

Here we present a user-friendly implementation of the HillCube technique suitable for large-scale networks in a MATLAB/Octave toolbox called *Odefy*. This software provides convenient access to different model sources, the conversion process itself and various analysis and export methods (Figure 1). Boolean models may be entered as sets of Boolean equations directly or created with the yEd graph editor [12]. The user may build conventional *interaction graphs *with activating and inhibiting edges or use an intuitive hypergraph representation of Boolean models [13]. In addition, models can be imported from the *CellNetAnalyzer *toolbox [6], GINsim [14] and the PBN toolbox [15]. After generating the ODEs, the user can easily adjust model parameters and perform time-course simulations using Odefy's graphical user interface. The ODE systems can be exported to MATLAB script files for further usage in MATLAB programs, to ODE script files for the R computing platform, to the SBML format, or to the well-established MATLAB Systems Biology Toolbox [16]. Due to the nice mathematical properties of the produced ODEs and the integration with state-of-the-art modeling tools, a variety of analysis methods can be immediately applied to the models generated by Odefy, including bifurcation analysis, parameter estimation, parameter sensitivity analysis and so on. For compatibility, we also integrated export options to the discrete model formats of the *Genetic Network Analyzer *[17] and *SQUAD *[18].

In this manuscript we first discuss the mathematical backgrounds and implementation details of the Odefy toolbox, including the different model import sources, analysis methods and export options. In the results section, two examples of quantitative modeling with our toolbox are given, namely a motif from stem cell differentiation and the regulatory network responsible for the establishment and stable maintenance of the mid-hindbrain boundary. We show the ease-of-use of the Odefy toolbox and demonstrate similar dynamical properties between a molecular model of the stem cell motif and the corresponding derived Odefy model. The mid-hindbrain example specifically emphasizes the importance of a fully automated conversion method from discrete to continuous models.

## Implementation

### Mathematical background

This section outlines the mathematical formulation of our automatic Boolean model conversion technique. For a detailed description of this methodology along with motivations, comparisons to similar approaches and application to a T-cell signaling model, we refer the reader to [8]. A Boolean model consists of *N *species *X*_1_, *X*_2_, ..., *X*_*N *_each taking a value *x*_*i *_∈ {0, 1}. The value of *X*_*i *_at time *t *+ 1 depends on the species *X*_*i*1_, *X*_*i*2_, ...,  and is given by the Boolean update function *B*_*i *_(*x*_*i*1_, *x*_*i*2_, ..., ) ∈ {0, 1}. In a discrete simulation, time is discretized and the values of *x*_1_, *x*_2_, ..., *x*_*N *_at time *t *+ 1 are determined by synchronously setting:

The main idea is to convert the above discrete model into a continuous ODE model, where species *X*_*i *_is allowed to take values  ∈ [0, 1], and its temporal development is described by the ordinary differential equation (ODE):

The right hand side of this equation consists of two parts, an activation function  describing the production of species *X*_*i *_and a first-order decay term. An additional parameter *τ*_*i *_is introduced to the system, which can be understood as the life-time of species *X*_*i*_.  can be considered a continuous homologue of the Boolean update function. The key point is how it can be obtained from *B*_*i *_in a computationally efficient manner.

In Odefy, three different methods to transform *B*_*i *_into  are implemented. They are shortly described in the following. For simplicity of notation, we omit the subscript *i*.

#### BooleCube

**Figure 2:**
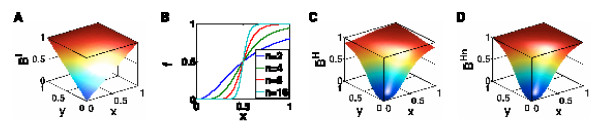
**Continuous homologues of Boolean functions**. Continuous homologues of Boolean functions. **A **Multilinear interpolation of a two-variable OR gate (Boole-Cube). **B **Hill functions with Hill coefficients *n *= 2, 4, 8, 16 and *k *= 0.5 as continuous relaxation of a Boolean step function. **C **Composition of BooleCube from (A) with Hill functions (HillCube). **D **normalized HillCube from (C).

The basis of all three transformation methods are the so-called *BooleCubes*:

which we obtain by multilinearly interpolating the Boolean function *B*, see Figure 2A.

#### HillCube

The functions  are affine multilinear. Many molecular interactions, however, are known to show a switch-like behavior, which can be modeled using sigmoid shaped *Hill functions *, see Figure 2B. The two parameters *n *and *k *have a clear biological meaning. The Hill coefficient *n *determines the slope of the curve and is a measure of the cooperativity of the interaction. The parameter *k *corresponds to the threshold in the Boolean model, above which one defines the state of a species as *on*. Mathematically speaking, it is the value at which the activation is half maximal, i.e. equal to 0.5. We now introduce a Hill function *f*_*i *_with parameters (*n*_*i*_, *k*_*i*_) for every interaction and define a new continuous function:

which we call *HillCubes*, see Figure 2C. We can show that for sufficiently large Hill exponents *n*, there will be a steady-state of the continuous system in the neighborhood of each Boolean steady-state [8].

#### Normalized HillCube

Note that Hill functions never assume the value 1, but approach it asymptotically. Hence, the HillCubes are not perfect homologues of the Boolean update function *B*. If this is desired a simple solution is to normalize the Hill functions to the unit interval. This yields another continuous (perfect) homologue of the Boolean function *B*:

which we call *normalized HillCube*, see Figure 2D.

### Implementation in MATLAB/Octave

The core functionality of Odefy is accessible through a set of functions for the MATLAB/Octave command line or via a Java-based graphical user interface. Figure 1 shows an overview of the complete Odefy tool-box. The following section provides detailed descriptions of the model definition and import process, ODE generation, model simulation and exporting.

#### Model definition & representation

**Figure 3:**
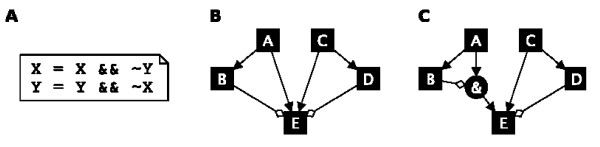
**Boolean model definition**. **A **The easiest way to define a Boolean model in Odefy is to specify a set of Boolean equations in a text file. This example represents an asymmetric version of the mutual inhibitory switch shown in the results section. Note the use of the MATLAB language-specific operators &&, || and ~. **B **Regulatory interaction graph created with the yEd graph editor. Regular arrows represent activatory influences whereas diamond-head arrows stand for inhibition. Note that we need to specify a generic logic to combine multiple regulatory inputs for node *E*. The Odefy default *at least one activator and no inhibitors *logic would result in *E *= (*A *∨ *C*) ˄ ¬ (*B *∨ *C*). **C **Alternative representation of the Boolean model as a hypergraph. Using a specialized node '&' we can precisely specify the Boolean logic for node *E*. All edges not incident to a '&' node are treated with an OR logic. The resulting Boolean update rule reads *E *= (*A *˄ ¬ *B*) ∨ *C *∨ ¬ *D*. ˄ = logical AND, ∨ = logical OR, ¬ = logical NOT.

An Odefy input model consists of a set of Boolean update rules for the underlying regulatory system. Our toolbox currently supports several possibilities to define such models:

(i) The user may enter a set of symbolic Boolean equations in text-form, allowing for the quick and intuitive generation of model structures (Figure 3A). Boolean equations consist of model variables and the three Boolean operators AND, OR and NOT. For the Odefy import process, we represent these operators by the MATLAB language-specific operators &&, || and ~, respectively. Throughout this manuscript, we stick to the common mathematical notation of ˄ for AND, ∨ for OR and ¬ for NOT.

(ii) Models can be derived from directed graphs created in the free yEd graph editing software [12]. The user builds an interaction graph of activating and inhibiting edges, which is then converted to an Odefy Boolean model (Figure 3B). Note that we need to specify how multiple regulatory inputs of a single factor are combined into a Boolean update rule. For this a generic logic of the form *f*(*X*) = (*A*_1 _⊖ *A*_2 _⊖ ... ⊖ *A*_*m*_) ⊙ ¬(*I*_1 _⊗ *I*_2 _⊗ ... ⊗*I*_*n*_) defined by three Boolean operators ⊖, ⊙, ⊗ ∈ {˄, ∨} is used, where *A*_1_, ..., *A*_*m *_is the set of activators and *I*_1_, ..., *I*_*n *_represent all the inhibitors of *X*. The Odefy default setting is to activate the output if at least one activator and no inhibitors are active. In order to create this behavior we choose ⊖ = ∨, ⊙ = ˄, ⊗ = ∨ resulting in

The assignment of Boolean operators can be changed during the import process into the Odefy toolbox. In addition to the possibility of inputing interaction graphs, we implemented an intuitive hypergraph-based representation of Boolean models in the *sum of product *form, which is capable of describing any Boolean update function [13] (Figure 3C).

(iii) Odefy can be tightly integrated with the well-established CellNetAnalyzer (CNA) toolbox [6]. By a plugin-like menu interface the user can execute Odefy from within CNA and convert existing CNA models into systems of differential equations. Furthermore, parameter settings made in the CNA user interface are directly passed to Odefy and used for simulation and exporting.

(iv) Finally, Boolean models can be directly imported from the GINsim XML format [14] and the Probabilistic Boolean Networks toolbox [15].

The Odefy toolbox can efficiently handle large-scale models containing 50 players and more. One of the largest cellular Boolean model, a T-cell model with 94 nodes and a total of 123 regulatory interactions [19], can be transformed and simulated in less than one second on a standard workstation. Internally, Boolean models are stored as multidimensional arrays (i.e. hypercubes with edge length 2) for rapid element access and Boolean function evaluation. The time complexity of model generation lies in (2^*N*^) with *N *being the highest degree of all nodes, yielding an exponential growth of computational runtime. The limiting size of Odefy models is thus not the number of nodes contained, but rather the highest number of incoming edges for any node in the model. For most regulated genes, however, we assume the number of modeled input regulatory factors to be equal to or less than 10, which can be handled on the order of one second per node by Odefy.

To account for systems consisting of multiple cells or, more generally, compartments driven by identical regulatory networks, Odefy contains an automated multicompartment expansion procedure. Given a Boolean model and the assignment of an *intercompartment *flag for a given set of factors in the model, Odefy generates a larger model corresponding to a linear row of connected compartments. Factors flagged as intercompartmental exhibit their influence towards the two neighboring cells and are combined using an OR logic (see also: Mid-hindbrain example below).

#### Simulation and analysis

**Figure 4:**
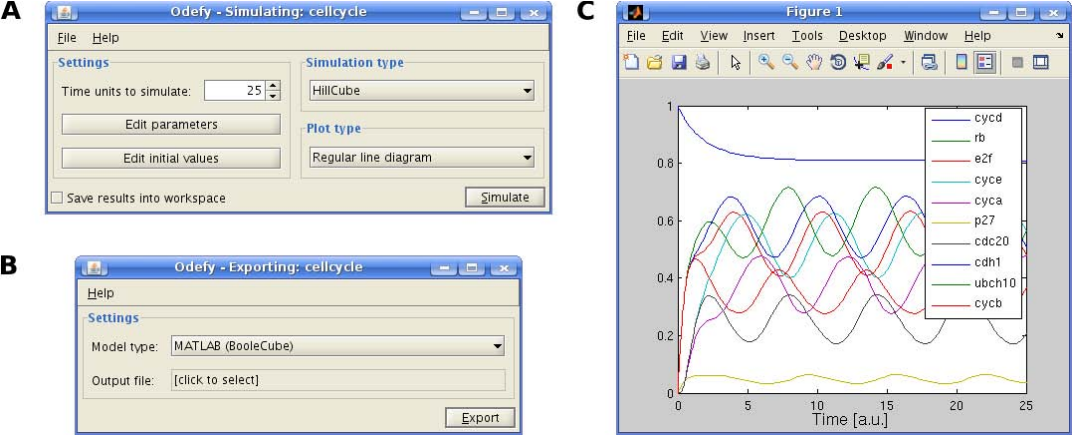
**Odefy graphical user interface**. **A **Screenshot of the Odefy simulation dialog for convenient access to the dynamic model parameters, initial values, conversion types and plot options. All settings can be saved to or loaded from the current workspace. **B **Export dialog for all discrete and continuous Odefy export formats. **C **Exemplary time-course simulation of the cell cycle model from [5] with default parameters.

After model creation, the resulting ODE systems can be simulated directly by numerical integration algorithms or, alternatively, exported to various external model formats. Note that Boolean models as such are parameter-free, and the dynamical parameters for the ODEs have to be set externally. For convenience, Odefy employs a set of reasonable default values for all parameters in order to allow for a quick analysis of the system. Import, parameter adjustment, simulation and exporting can be accessed by the Odefy command line functionality as well as a graphical user interface (Figure 4). These Java Swing-based user dialogs provide a platform-independent look and feel. They use the MATLAB-internal Java engine and therefore do not require an external Java runtime environment. For advanced MATLAB users and users of the Octave environment, we provide functions for convenient parameter access, Boolean state analysis (steady states and state-transition graph) and phase plane visualization of dynamic simulations.

#### Export

Export formats for Odefy models include the MATLAB/Octave ODE script files, the Systems Biology (SB) Toolbox [16], the SBML format, script files for the R computing platform, the *Genetic Network Analyzer *(GNA) [17] and *SQUAD *[18]. SB Toolbox contains various advanced analysis functions for dynamical systems like parameter sensitivity and bifurcation analysis. The SBML format can be read by various systems biology software tools like COPASI [20] and CellDesigner [21] and thus provides a versatile interchange format. The GNA allows a structural analysis and qualitative simulations of systems of piece-wise linear ODEs. SQUAD analyzes discrete and continuous models using the standardized qualitative dynamical systems approach.

#### Toolbox

The Odefy toolbox is platform-independent due to the availability of MATLAB and Octave for all major operating systems and the direct integration of the Java Runtime Environment into MATLAB. It was verified to run smoothly on Windows, Linux KDE and GNOME as well as recent versions of Mac OS X. A detailed HTML documentation is included in the download package, which also provides a quick start guide to start working with the toolbox. Odefy is free for non-commerical and academic use. The toolbox including source codes can be downloaded at http://cmb.helmholtz-muenchen.de/odefy.

## Results and Discussion

### Mutual inhibitory switch

**Figure 5:**
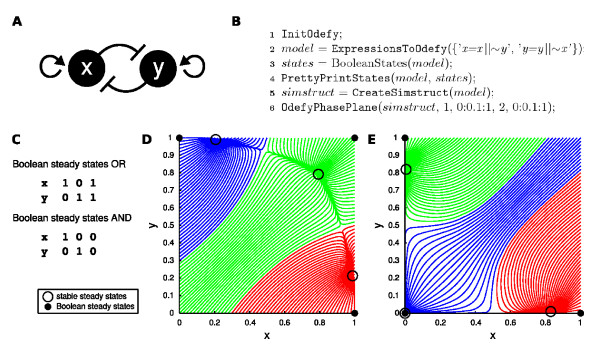
**Mutual inhibitory switch**. **A **Regulatory network known to take a prominent role in stem cell differentiation processes (see e.g. [22]). It consists of two mutual inhibitory factors (here with auto-activation). Intuitively, only one of the two factors can be fully active at any given time, leading to a switch-like behavior of this circuit. **B **This listing diplays the set of commands required to create and analyze the OR logics version of the mutual inhibitory switch. After initializing Odefy and generating the model structure (lines 1-2), we calculate and output Boolean steady states (lines 3-4) and finally convert the model into an ODE system to generate a picture of the attractor landscape (lines 5-6). **C **Boolean steady states of the OR and AND version of the mutual inhibitory switch model. **D, E **Phase planes visualizing the attractor landscapes of the OR (**D**) and AND (**E**) logics models. The figures display trajectories of both dynamical systems from various initial concentrations. Trajectories with the same color fall into the same stable steady state. Both systems comprise three stable continuous steady states, each of which belongs to one Boolean steady state.

In the following we demonstrate the use of Odefy for the analysis of a simple regulatory motif. The mutual inhibitory switch (Figure 5A) is a well-known circuit involved in developmental processes and stem cell differentiation, e.g. in the hematopoietic system [22]. Despite its simplicity the circuit displays remarkable dynamic characteristics leading to the fate decision between opposing differentiation lineages. Various theoretical studies have been published recently investigating different aspects and molecular assumptions for this motif [23-25]. We discuss two different ways of formulating the interactions in this network in terms of Boolean equations. Multiple regulatory inputs (in this case self-activation and cross-inhibition) can either be combined using an AND or an OR logic for both factors. Figure 5B shows the MATLAB code that analyzes the OR logics version of our mutual switch network. After creating the model structure we calculate and output the steady states of the Boolean model (Figure 5C).

We demonstrate the actual conversion into an ODE model and subsequent simulation within the Odefy toolbox. A two-dimensional phase plane projection of various initial values is drawn that displays the attractor landscape generated by the dynamical system (Figure 5D, the phase plane visualization for the corresponding AND logics model is shown in Figure 5E). Note that this analysis reveals continuous decision boundaries between different attractors not apparent in the discrete model alone. Furthermore, two unstable steady states emerge which mark the switching points from one attractor basin to the other. In stem cell research, the central state is considered to be a pre-differentiation *priming *state whereas the other two states correspond to the regulatory program leading to the commitment to a certain cell lineage [26]. With our continuous mathematical representations we gain insights into the putative switching dynamics of this important differentiation switch in stem cells. After fitting simulated trajectories to observed time series of expression data, we could now determine rate parameters and understand the detailed time dynamics of the system.

### Comparison with an existing ODE model

**Figure 6:**
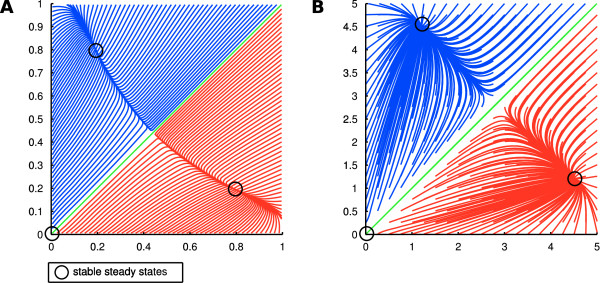
**Comparison with an existing modeling study**. **A **Phase planes visualizing the attractor landscape of the Odefy-converted AND version of the mutual inhibitory switch from various initial conditions. We set the Hill parameter *n *to 2 in order to represent dimer binding of transription factors as proposed in the study by Roeder et al [23]. The self-activation threshold *k*_*s *_was set to 0.01, resembling a highly sensitive self-activation in comparison to the mutual inhibition. **B **Simulation of the ODE system from [23] with a high *unspecific transription rate*. We show an exact reproduction of the phase plane displayed in Figure 2(h) from the original publication. Both dynamical systems are similar in terms of multistability, steady state positions and attractor basins, i.e. the initial values that fall into a certain steady state.

We now employ the mutual inhibitory switch model discussed in the preceding section to address an important question for our novel modeling approach, namely whether the quantitative dynamics added to the discrete model are reasonable, or whether spurious, artifical effects are created by the method. In the study by Roeder et al. [23], a mechanistical model of the switch motif was proposed, which is based on actual biochemical reactions like promotor binding, transcription/translation and protein-protein interactions. The system was reduced to a two factor ODE by applying quasi-steady assumptions for the DNA and RNA species in the system. A comparison between simulation trajectories of the Odefy-converted model of the AND-gated switch and the Roeder model is displayed in Figure 6. Both systems have two non-zero stable steady states at similar positions, and the attractor basins for both states are virtually identical. Furthermore, both systems comprise a third, trivial steady state where both factors are zero. Interestingly, the parameter assignments we made for the simulation of the Odefy model, in order to achieve similarity between the model simulations, is qualitatively comparable with the parameter settings from the Roeder model. More precisely, the Roeder model employs a high *unspecific transcription rate *(we refer to the original publication for more details on the parameters), which effectively reduces the mutual inhibitory influences in relation to the autoregulatory activation of both factors. Accordingly, in our model we set the self-activation threshold to 0.01, which renders both factors strongly sensitive to their own expression levels. Taken together, we can reproduce important dynamical features of the reaction-based system by Roeder et al., inluding multistability, steady state positions, and the general shape of the attractor basins.

### Mid-hindbrain boundary

**Figure 7:**
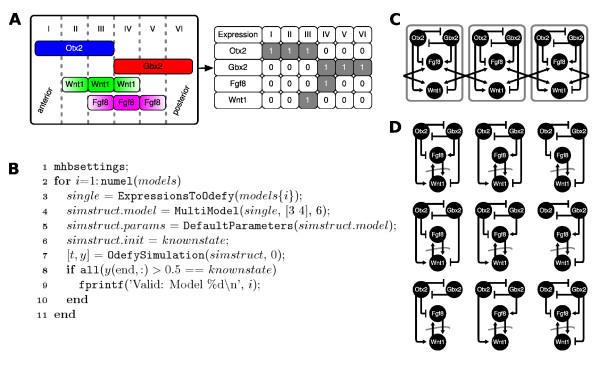
**The Mid-hindbrain boundary**. **A **Expression patterns of four major factors at the mid-hindbrain boundary. The relevant part of the neural tube is subdivided into 6 compartments, each displaying a unique expression pattern. The right table represents the known expression patterns in a Boolean framework (note that the secreted variants of Fgf8 and Wnt1 are not included here). **B **This code snippet demonstrates the use of Odefy for model selection. We load a precalculated set of random models along with the 9 known valid models and iterate over all model equations (lines 1-2, mhbsettings is contained in the examples folder of the Odefy package). Next, we generate Boolean models from each equation and extend the model to a six cell multicompartment model (lines 3-4). The Boolean model is converted into a system of ODEs and simulated starting from the known expression state (lines 5-7). Note that we simulate a sufficient amount of time units to ensure the systems has fallen into a steady state. If the final state after simulation is still correct in terms of activity we assume to model to be valid (lines 8-10). **C **Three replicated cells from our hypothetical six-cell system. Note that Wnt1 and Fgf8 are secreted factors and exhibit their influence towards neighboring cells. **D **The nine network variants that give rise to the desired steady state pattern. All models contain the mutual activation between Fgf8 and Wnt1 (between neighboring cells), a mutual inhibition between Otx2 and Gbx2 as well as some regulatory incluence of the transcription factors towards the secreted factors.

Our second example of dynamic modeling using Odefy concentrates on a multicellular biological system. During vertebrate development, the differentiation between mid- and hindbrain is determined by several transcription factors (e.g. Otx2, Gbx2) and secreted factors (e.g. Fgf8, Wnt1). These genes are stably expressed in a well-defined spatial pattern around the boundary between prospective mid- and hindbrain, the so-called mid-hindbrain boundary (Figure 7A). In a recent publication, we have applied both Boolean modeling and the HillCube conversion approach to this system [27]. In the following we will show how to use Odefy for automated model selection, that is the evaluation of an ensemble of regulatory networks with respect to stability of the known expression patterns. Figure 7B displays the MATLAB code required to fulfill this goal. First, we load a set of 9 regulatory networks known to give rise to the expression pattern along with 1000 random Boolean equations (not shown in the code) as a representative set of arbitrarily chosen regulatory networks. Then we iterate over all equation systems, generate a 6 cell multicompartment version of this model where the species representing the signaling molecules Fgf8 and Wnt1 are flagged as intercompartmental (Figure 7C). The multi-cell system is converted to an ODE system and simulated starting from the known stable expression state with default parameters *n *= 3, *k *= 0.5, *τ *= 1. If the activity of all players, in terms of exceeding the Hill threshold parameter, is still correct after a given amount of time steps, we consider the model to be valid. The results of this experiment show that indeed only 9 networks can give rise to the desired system behavior (Figure 7D). Analyzing these networks we see, in particular, that the maintenance of the boundary requires a mutual inhibition of Otx2 and Gbx2 and that these two transcription factors have antagonistic effects on Fgf8 and Wnt1 expression. Moreover, we find that Fgf8 and Wnt1 require each other for their stable maintenance. This agrees well with results from various loss-and gain-of-function experiments [28]. Note that while the small network in the former sections could still be handled manually, the model selection problem for the mid-hindbrain network demonstrates the absolute necessity for fully automated approaches as implemented in our toolbox. The system contains 6·4 = 24 differential equations with a total of up to 20 kinetic parameters for each compartment. Obviously, a model system of this size with parameter interdependencies due to multicompartmentality cannot reasonably be handled by manual mathematical modeling.

## Conclusions

Precise mechanistic details about regulatory interactions required for the quantitative modeling of biological systems are rare. However, more qualitative, phenomenological information like *activation *and *inhibition *is frequently available. With Odefy we created a simple yet useful toolbox to bridge the gap between qualitative and quantitative modeling of regulatory networks. A variety of such discrete models is already available and can immediately be converted into ODE systems by our tool.

Quantitative modeling might reveal features not present in the original Boolean models. For instance, quantitative models allow for the estimation of system robustness with respect to parameter perturbations, even with ad-hoc parameter values. This provides insights into the general capability of the system to withstand external or intracellular fluctuations and has been demonstrated for various biological systems like Drosophila segmentation patterns [29] and the mid-hindbrain specification mentioned in this report. Furthermore, in [8] we determined parameter values by least-square fitting to experimental data in a T-cell signaling model. We could, amongst others, successfully predict relations between binding affinity constants of ligand-receptor interactions, which represent biochemical quantities not capturable in a Boolean framework.

In this report we explained the concepts of automatic conversion from Boolean models to systems of ordinary differential equations. Two example cases were discussed stressing (a) the ease-of-use of the Odefy toolbox as well as (b) the requirement for automated conversion methods for more realistic biological systems like the Mid-hindbrain boundary network. We demonstrated that a discrete model converted to an ODE by Odefy displays similar dynamical properties as a mechanistically derived ODE model of the same system. Here we could show that, even though the identity of dynamical parameters between both modeling approaches is substantially different, qualitatively similar parameter changes show similar results.

The integration of Odefy with other modeling applications through the import and export of models extends the scope of our toolbox. In particular, the SBML export functionality connects our toolbox to a broad variety of systems biology softwares supporting this common interchange format. With its novel modeling technique and its easy usability, Odefy will be a valuable tool for researchers aiming to understand the dynamics of gene regulation.

## Availability and requirements

• **Project name**: Odefy

• **Project home page**: http://cmb.helmholtz-muenchen.de/odefy

• **Operating system(s)**: Platform independent

• **Programming language**: MATLAB/Octave

• **Other requirements**: MATLAB 7.1 or higher (no additional toolboxes required), Octave for non-GUI mode

• **License**: Free for non-commercial purposes

## Authors' contributions

DMW and FJT developed the automatic conversion technique and carried out the Mid-hindbrain study. JK and SP developed the Odefy application. JK and DMW wrote the initial manuscript. All authors revised and approved the final manuscript.
